# Genome-wide DNA methylation analysis reveals different methylation patterns in Chinese indigenous sheep with different type of tail

**DOI:** 10.3389/fvets.2023.1125262

**Published:** 2023-05-05

**Authors:** Zhu Caiye, Shuzhen Song, Minna Li, Xaioyu Huang, Yan Luo, Suli Fang

**Affiliations:** ^1^College of Animal Science and Technology, Gansu Agricultural University, Lanzhou, Gansu, China; ^2^Institute of Animal and Pasture Science and Green Agriculture, Gansu Academy of Agricultural Sciences, Lanzhou, Gansu, China; ^3^Gansu Institute of Animal Science and Veterinary Medicine, Pingliang, China

**Keywords:** sheep, tail type, tail fat deposition, DNA methylation, genome

## Abstract

**Background:**

The study was aimed to analyze the difference of genome-wide DNA differential methylation in Lanzhou Large-tailed sheep, Altay sheep and Tibetan sheep, which the typical breeds with different type tails, as to screen the differentially methylated genes (DMGs) that affect the type of tails.

**Methods:**

In this study, three Lanzhou Large-tailed sheep, three Altay sheep and three Tibetan sheep were detected by whole genome bisulfite sequencing (WGBS). The degree of genome-wide DNA methylation, differentially methylated regions (DMRs) and DMGs were analyzed. The candidate genes affecting the tail type of sheep were identified by GO and KEGG pathway enrichment analysis of DMGs.

**Results:**

we identified 68,603 different methylated regions (DMCs) and 75 differentially methylated genes (DMGs) associated with these DMCs. Functional analysis showed that these DMGs were mainly enriched in biological process, cellular component and molecular function, Some of the genes in these pathways are involved in fat metabolism: *NFATC4*, *LPIN2*, *MGAT2* and *MAT2B*.

**Conclusion:**

Our results may help to further understand the epigenetic regulation mechanisms of deposition of fat in the tail of sheep and provide new basic data for the study of local sheep.

## Introduction

Fat deposited on the tail of a sheep is an adversity biological trait, Its role is manifested in two aspects: (1) fat as a form of energy storage, can provide energy for sheep to migrate or survive the cold winter lack of forage, so as to maintain their survival needs (1); (2) in drought and In the famine years, fat at the tail of sheep can be used as a high-energy food for human use (2). Fat-tailed sheep are derived from wild thin-tailed sheep through artificial domestication and natural selection (3). In the process of domestication and selection, the climatic environment and human needs and different selection methods will have an important impact on the formation of different tail types. Most fat in Lanzhou Large-tailed sheep and Altay sheep is deposited in the tail, which will reduce the deposition of fat in other parts of the carcass, which will affect meat quality (4), moreover, the fat needs more feed than meat production. Therefore, tail fat deposition reduces the benefits of sheep farming, and studying the molecular mechanism of sheep tail fat deposition has important scientific significance and broad application prospects in breeding small-tail low-fat sheep breeds.

In mammals, DNA methylation refers to the supply of methyl donors with S-adenosylmethionine (SAM) under the action of DNA-methyl transferase (DNMT), Transfer its methyl group to the 5th carbon atom of the deoxycytosine ring to form a covalent modification of methylated deoxycytosine (5mC) (5). 5mC generally appears on the cytosine of CpG, CpG sites can account for 5–10% of the mammalian genome. The methylation status of CpG is closely related to gene expression, DNA methylation can inhibit the activity of the accessory gene, and demethylation can induce gene re-expression. Phenotypic differences are not entirely explained by genetic differences, studies have shown that DNA methylation can explain phenotypic differences, such as the phenotypic difference of twins, cloned animals (6–8). DNA methylation regulates the growth and development of adipose tissue mainly by regulating the expression of transcription factors related to adipocyte differentiation, transcription cofactors and genes related to fat metabolism (9). Zhang have shown that methylation of the gene promoter region may inhibit the expression of genes related to fat metabolism, thereby affecting lipid droplet structure and fat deposition (10).

The tail type of sheep is a complex trait, which was affected or interacted by genetic and environmental factors. DNA methylation plays an important role in growth and gene expression in sheep. Mo correlation analysis re studies have shown that DNA methylation can explain phenotypic differences. Chinese indigenous sheep can be classified into fat-tailed, fat-rumped and thin-tailed sheep, of which the typical breeds are Lanzhou Large-tailed sheep, Altay sheep and Tibetan sheep, respectively. To unravel the phenotypic differences among different type tails, in this study, MeDIP-seq was used to perform genome-wide DNA methylation association analysis on three breeds of Lanzhou large-tail sheep, Altay sheep and Tibetan sheep, in order to finding candidate differential methylation regions or sites that potentially affect tail fat deposition, and provide a mechanism for revealing fat deposit mechanisms.

## Materials and methods

### Ethics statement

All of the animal procedures were performed in strict accordance with the guidelines proposed by the China Council on Animal Care and the Ministry of Agriculture of the People’s Republic of China. All of the animal experiments were approved by the Gansu Agricultural University (Lanzhou, China), approval No. GSAU-AEW-2017-0003.

### Sample collections

**Figure 1:**
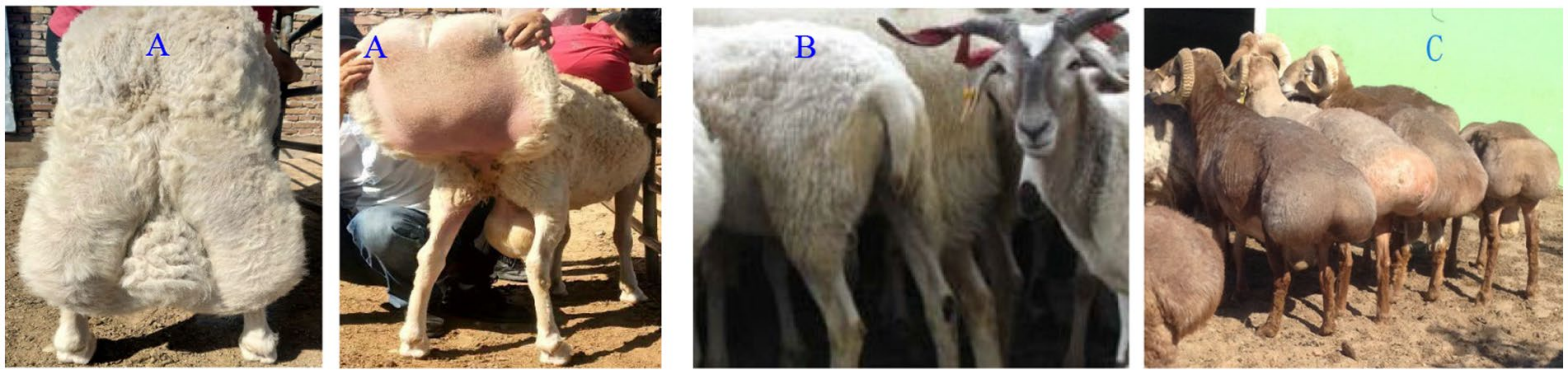
**(A)** Lanzhou Large-tailed sheep with fat-tail. **(B)** Tibetan sheep with thin-tail. **(C)** Altay sheep.

Nine individuals from 3 breeds, all are 1 year old rams, including 3 Lanzhou Large-tailed sheep, 3 Altay sheep and 3 Tibetan sheep, were collected from Yongjing in Gansu Province, Fuhai in Xinjiang Province and Tianzhu in Gansu Province, respectively. All of the individuals were selected with the same sex, weight and age for the same tail type, and there is no genetic relationship between individuals. The tail length, width and circumference of Lanzhou big-tailed sheep are 38, 32, and 106 cm, respectively. The tail length, width and circumference of Altay sheep are 22, 36, and 98 cm respectively, The tail length, width and circumference of Tibetan sheep are 15, 4, and 7 cm, respectively (Figure 1).

Take fresh blood from the jugular vein in the EDTA vein vacuum blood collection tube and store it in the refrigerator at −80°C. Genomic DNA was extracted from the blood samples of 9 individuals using Tiangen blood genomic DNA extraction kit (DP318, Purchased from Beijing Tiangen Biochemical Technology Co., Ltd.). Then through the Nano-Drop spectrophotometer and gel electrophoresis test, reserve after being qualified.

### Establishment of MeDIP-seq sequencing library

Genomic DNA ultrasound interrupted to 100–500 bp fragment, DNA fragment end repair add A base at 3’end, connect to sequencing adapter, which were treated by bisulfite conversion with EZ DNA Methylation-Gold kit (Zyom Research Corporation, Irvine, CA, USA). Cut and recycle after desalting and select the library fragment size, qualified libraries were used for computer sequencing. HiSeq2000 (Illumina, San Diego, CA, USA) was used to send to Benatech for library establishment and sequencing.

### Bioinformatics analysis

After the sequencing is completed, the sequencing adapters and low-quality data are filtered, the filtered data is compared with the reference genome, and the sequencing result is compared with the reference genome, and the sequence at the only position in the comparison is used for subsequent standard information analysis and personalized analysis. The data filtering criterion are: contains adapter sequences, the proportion of N bases in the sequence exceeds 5% of the total length of the sequence, and the proportion of bases with a quality value less than 20 in the sequence exceeds 50% of the total length of the sequence, if a sequence meets the above three conditions any one of them, then remove this sequence. The data comparison parameters are: BSMAP software is used for comparison, and the comparison mode is map to 2 forward strands, i.e., BSW(++) and BSC(−+).

### Calculate the methylation level of the C base

The methylation level of each methylated C base is calculated according to the following formula: Methylation rate at C site = 100* Reads that support methylation/(Reads that support methylation+ Support unmethylated reads). The sites with a sequencing depth greater than 3 are judged as reliable methylation sites, and use Bedtools software to map the methylation sites of different gene elements in each sample (11).

### Plotting genome-wide DNA methylation maps

According to the average methylation level of each 100 bp interval, draw the methylation map of the gene body or both sides of the transposable element in the 2Kb region to evaluate the methylation patterns of different genomic regions (12, 13).

### Differentially methylated region (DMR) analysis

To determine the methylation types (CpG, CHG and CHH) in the different regions between the 3 species, according to the following strict standards: (1) Find a window containing at least 10 C bases at the same position in the genome of the three samples, (2) Each cytosine site is greater than 10 reads, and each methylated cytosine is greater than 4 reads, (3) The region length ranges from 40 bp–10 Kb, (4) The distance between adjacent methylation sites is less than 200 bp, (5) The average methylation level multiple is greater than 2 and *P*-adjust <0.05. Find the region of differential methylation between different breeds in the adjacent 2 Kb (upstream or downstream), transposon or gene main region.

### Differentially methylated gene enrichment analysis

Studies have shown that methylation mainly regulates gene expression through gene promoter regions. Select genes with DMR in the differentially methylated gene promoter (upstream 2 kb region) for gene function GO and signal pathway KEGG enrichment analysis, and select GO/KEGG Term, the results of gene number > 1, enrichment multiple > 2, value of *p*<0.05 (part of the results could not be screened) were plotted.

## Results

### Date information

The comparison results are shown in Table 1. In Lanzhou Large-tailed sheep, a total of 517,022,996 reads were obtained. The number of reads compared to the sheep genome was 515,987,112, with an average comparison rate of 99.80%. In Altay sheep, total of 523,543,986 reads were obtained and the number of reads compared to the sheep genome was 471,216,836, with an average comparison rate of 90.20%. A total of 529,964, 194 reads were obtained in Tibetan sheep, with a clean rate of 99.80%.

**Table 1:** Results of genome comparison using BWA software.

Sample	Raw reads	Raw bases	Clean reads	Clean rate
Lanzhou Large-tailed sheep	517,022,996	77,553,449,400	515,987,112	99.80%
Altay sheep	523,543,986	78,531,597,900	471,216,836	90.20%
Tibetan sheep	529,964,194	79,494,629,100	528,891,456	99.80%

### Comparison of genome-wide methylation rates

#### Distribution of methylation sites in the whole genome

There are three main patterns of DNA methylation at CpG sites (CG, CHG and CHH) in the genome, and the number and composition ratio of these three patterns reflect the characteristics of genome-wide methylation in a given species. The average methylation rate of the whole genome of the three breeds is shown in Table 2. Among the three sheep breeds with different tail types, the CG type has the highest methylation rate, and the average value of the three breeds is 66.16%, which is significantly higher than that of the CHG and CHH types. The distribution of methylation levels in the Table 2 shows that three different tail types of sheep have the highest overall CG background methylation levels, while CHG and CHH background methylation levels are extremely low or even close to zero.

**Table 2:** Methylation levels in CG, CHG and CHH.

Sample	C	CG	CHG	CHH
Lanzhou Large-tailed sheep	3.62%	64.57%	0.63%	0.65%
Altay sheep	3.67%	65.53%	0.61%	0.63%
Tibetan sheep	3.74%	68.40%	0.59%	0.61%
Average	3.67%	66.16%	0.61%	0.63%

#### Analysis of the methylation level of gene functional regional in the whole genome

**Figure 2:**
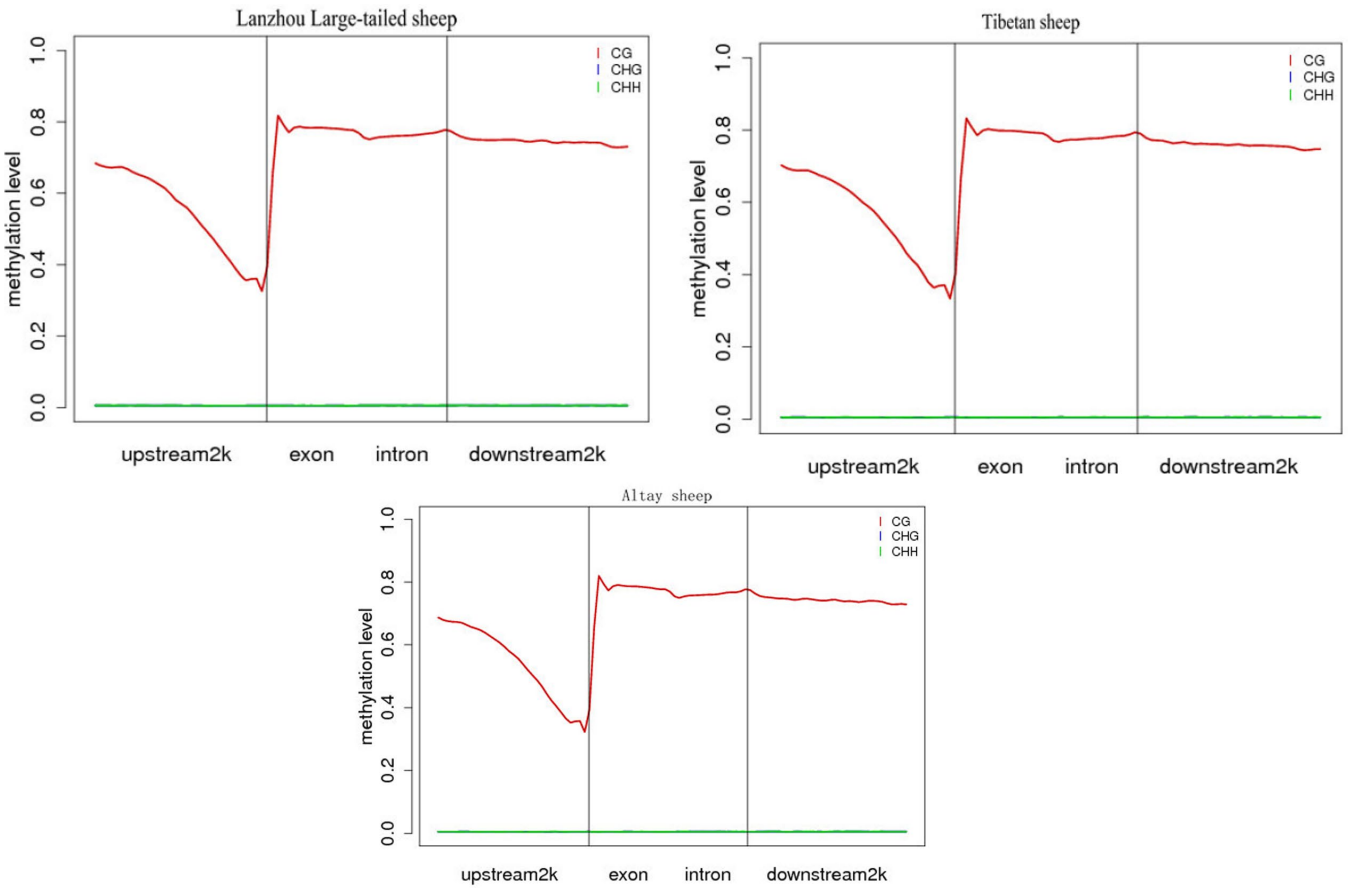
Distribution of methylation levels in different functional element regions of the whole country group.

The analysis showed that CG pattern methylation accounted for most of overall methylation. To be better understanding the distribution trend of DNA methylation in the CG context of genomic regions, we counted the DNA methylation levels on six functional regions and seven transcription elements, including upstream2kb, exon, intron, 3-UTR, downstream 2Kb and intergenic regions. The results showed that the methylation levels of different functional regions and transcription elements were significantly different (Figure 2). Among the functional regions, the 5’UTR region had the lowest methylation level, the 3’UTR and Gene region had the highest average methylation level, and among the transcription elements, the First exon had the lowest methylation level, and the Internal exon, Internal intron, and Last exon regions had the highest methylation level. However, the differences in methylation levels among different samples in the same functional region or transcriptional element were not significant.

#### Differentially methylated region (DMR) analysis

Find a window containing at least 10C bases at the same position in the genome of the three samples, and use the difference in C methylation levels to find regions with differences in methylation. The statistical results (Table 3) show that the significant DMRs in the CG environment of Altay sheep and Tibetan sheep were 60,631, the significant DMRs in the CG environment between Altay sheep and Lanzhou Large-tailed sheep were 68,603, between Lanzhou Large-tailed sheep and Tibetan sheep were 65,819.

**Table 3:** Number and length of DMR.

Group	Number of DMR	Number of cytosine	Length of DMR region
ALT108_vs_LZL13	68,603	815,809	27,384,493
ALT108_vs_Z204	60,631	722,624	24,297,767
LZL13_vs_Z204	65,819	776,870	26,097,332

**Figure 3:**
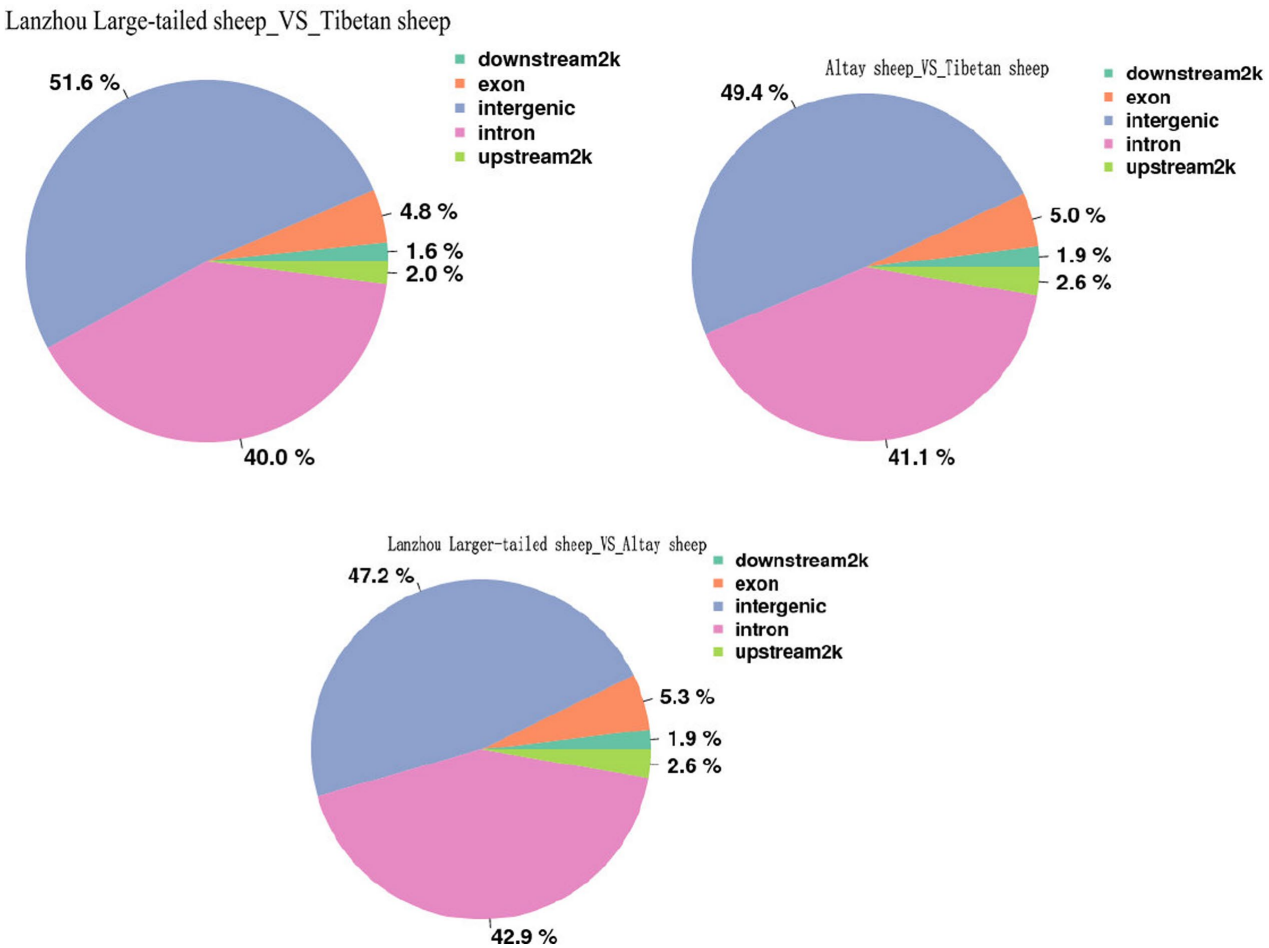
Distribution of DMR in different functional element areas.

In this study, the DMRs identified above were annotated using the annotation information of functional elements in the Ovis aries genome. Annotate DMR to gene elements (upstream2k, exon, intron, 3-UTR, downstream 2 k, intergenic), and count the distribution of DMR on gene elements. Among them, the most DMRs were found in intron regions (47.2–51.6%), followed by exon regions (4.8–5.3%), and DMRs in other regions were relatively low, only 2.0% of DMRs are distributed in the promoter region, and the methylation rate of the promoter region is lower than the gene body region (Figure 3).

#### Functional enrichment analysis of differentially methylated genes

DAVID (version 6.8),^1^ was used to conduct the GO and KEGG pathway enrichment analyses to investigate the functions of the candidate genes. After this enrichment analysis, followed by the Benjamini correction procedure, there were 20 GO entries were significantly enriched (*p* < 0.05), and 2 KEGG pathways were significantly enriched (*p* < 0.05).

**Figure 4:**
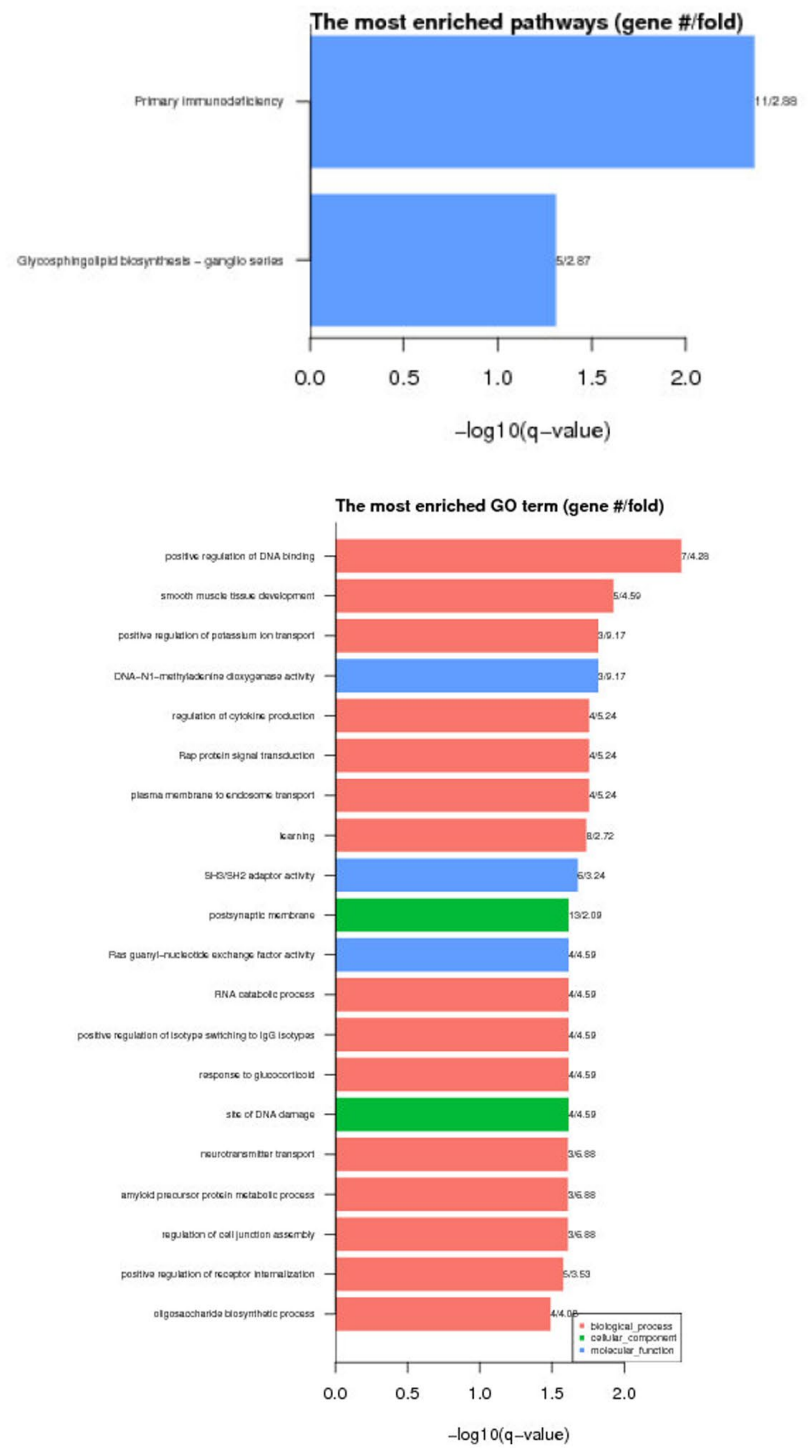
Functional enrichment results of differentially methylated genes.

GO results show that these differential genes are mainly related to molecular functions, cell components and biological processes. KEGG enrichment results show that these differential genes are mainly involved in Vitamin B6 metabolism Glycosylphosphatidylinositol (GPI)-anchor biosynthesis, the results are shown in Figure 4.

Combined with genome-wide DMR mapping and DMG function analysis, it was found that most of the genes enriched by GO and KEGG were located in intron regions, and a few were located in Promoter, 5’UTR, and Exon regions. Among the DMR, we found some genes related to fat synthesis and metabolism (Table 4).

**Table 4:** Fat synthesis and metabolism genes.

Gene name	*p* value	Functional description
*NFATC4*	0.022	Intramuscular fat deposition (14)
*LPIN2*	0.024	Related to fatty liver (15)
*MGAT2*	0.042	Fat deposition (16)
*MAT2B*	0.033	Regulates fat metabolism (17)

## Discussion

In the process of sheep domestication, traits that can adapt to the environment and increase energy production will be selected. Under the action of artificial selection, traits related to environmental adaptability and production performance will be further fixed in the genome, and then different breeds will be formed. The epigenetic modification induced by environmental factors will also have a direct impact on some traits of sheep (18). DNA cytosine methylation is an important epigenetic modification, which plays an important role in the regulation of gene expression, development and disease (12).

The main production area of Lanzhou Larger-tailed sheep is located in the suburbs of Lanzhou City. The production area is located in the northwest of the Loess Plateau, with an altitude of 1,500–2,500 m. The winter is longer and the temperature is 9.5°C, the growing season is short, and the sunshine is abundant. The central production area of Altay sheep is in Fuhai County, Xinjiang. The production area is located in the middle mountain belt of the Altai Mountains, with an altitude of 350–3,200 m. It belongs to a typical continental climate. The annual average temperature is 4°C, the extreme minimum temperature is −42.7°C, and the frost-free period is 147 days. The annual snow cover period is 200–250 days. Tibetan sheep are native to the Qinghai-Tibet Plateau and are mainly distributed in Tibet, Qinghai and Gansu. The average altitude is 4,000 meters. It is known as the “roof of the world.” The climate is characterized by long sunshine, strong radiation, low temperature, large temperature difference, and winter The spring is dry, the oxygen content is low, and the annual average temperature is −2.8-11.9°C (19). Numerous studies have shown that the interaction between genes and the environment can be explained by DNA methylation. The environment affects gene expression by affecting the level of DNA methylation, thereby changing the phenotype of organisms. The variety we chose is in a different environment and has different tail types. We will explore whether the environment causes methylation, which in turn causes different tail types.

DNA methylation is one of the main forms of epigenetic regulation, which plays an important role in the regulation of gene expression and the maintenance of genome stability (10, 20–22). DNA methylation is caused by the environment. These breeds we selected are in different environments and have different tail types. We will explore whether the environment causes methylation, which in turn causes different tail types. Previous studies have shown that methylation of the CpG site in the promoter region of a specific gene is involved in lipid metabolism (23).

In this study, we performed a genome wide DNA methylation analysis among Lanzhou big-tail sheep, Altay sheep and Tibetan sheep. The geographical environment of these three varieties is completely different. We identified 2,015,487 CpG sites, the methylation level of the CG type accounted for the largest proportion, while that of the CHH and CHG types was the smallest, even close to 0. A large number of studies have also confirmed that the DNA methylation pattern of mammals is dominated by the CG types (24). Our results found on average 66.16% of methylation occurs in the CG environment, this is consistent with the research result of others (25). It is suggested that the DNA methylation pattern has a certain degree of conservation.

We identified the significant DMRs in the CG environment of Altay sheep and Tibetan sheep were 60,631, the significant DMRs in the CG environment between Altay sheep and Lanzhou Large-tailed sheep were 68,603, between Lanzhou Large-tailed sheep and Tibetan sheep were 65,819. Among them, only 2.0% of DMRs are distributed in the promoter region, most of the DMRs are distributed in the intergenic region and intron region, and the methylation rate of the promoter region is lower than the gene body region. This may be related to the large proportion of intergenic regions and intron regions in the entire genome. The percentages of DMCs annotation within promoter and exon region decreased dramatically, while DMCs annotation within intron and intergenic regions increased when comparing DMCs with CpG sites annotation within genic features. And similarly, the percentages of DMCs annotation within CpG island shores and other regions increased, whereas DMCs annotation with CpG islands decreased. Wang et al. also found a similar trend in their study, which is consistent with this study (26, 27). Among the transcription elements, the methylation rate in the internal exon, internal intron, and last exon regions is the highest overall. Methylation patterns similar to the above are also very common in other mammals (28). Although the number of differential methylation regions screened from these three breeds were differed greatly, that three groups of DMR mainly annotated Intron and Exon regions, respectively. Studies have shown that the DNA methylation of Exons and Introns is relatively stable, showing a weak positive and negative correlation with gene expression (29). Introns, which are closely related to gene expression. However, due to the large scale of differential methylation regions in introns, it is difficult to screen functional differential methylation genes.

Based on functional annotation analysis, we found that these DMGs were mainly enriched in positive regulation of DNA binding, smooth muscle tissue development, DNA-N1-methyladenine dioxygenase activity, et al. These results confirmed our hypothesis that Lanzhou Large-tailed sheep, Altay sheep and Tibetan sheep would have epigenetic differences that reflect their specific phenotypic traits among the three breeds. These DMCs and DMGs contribute to the in-depth analysis of molecular genetic mechanism of sheep with different type of tail, and will be given more attention in epigenetic analysis of complex traits for Lanzhou Large-tailed sheep, Altay sheep and Tibetan sheep.

In this study, four tissue-specific candidate genes were screened, including *NFATC4*, *LPIN2*, *MGAT2* and *MAT2B*. *Lipins* play dual function in lipid metabolism by serving as phosphatidate phosphatase and transcriptional co-regulators of gene expression, Jiao et al. (15) reported that *Lpin2* and *Lpin3* mRNA expression both showed significant correlations with slaughter and tail traits. Taisuke et al. reported that *MGAT2* may have potential for development into a treatment of obesity and its related metabolic diseases (16). Zhao et al. reported that *MAT2A* promotes porcine adipogenesis by mediating H3K27me3 at Wnt10b locus and repressing Wnt/β-catenin signaling (30).

## Conclusion

This study performed epigenome-wide DNA methylation of tail fat tissue in Lanzhou Large-tailed sheep, Altay sheep and Tibetan sheep using MeDIP-seq sequencing technology. The results showed that the methylation pattern of sheep with different type were dominated by CG type and the differential methylation region was mainly located in introns and exons. Four annotated genes were associated with fat metabolism (*NFATC4, LPIN2, MGAT2* and *MAT2B*). Our findings provide new insights into a better understanding of the epigenetic regulation of fat deposition in sheep tail and will be useful to understand tail type traits in sheep, and thus contribute to breeding of thin-tail sheep as molecular marker.

## Data availability statement

The datasets presented in this study can be found in online repositories. The names of the repository/repositories and accession number(s) can be found in the article/supplementary material.

## Ethics statement

All of the animal procedures were performed in strict accordance with the guidelines proposed by the China Council on Animal Care and the Ministry of Agriculture of the People’s Republic of China. All of the animal experiments were approved by the Gansu Agricultural University (Lanzhou, China), approval No. GSAU-AEW-2017-0003.

## Author contributions

ZC conceived and designed the experiments, performed the experiments, and wrote the paper. SS, ML, XH, YL, and SF analyzed the data and contributed reagents, materials, and analysis tools. All authors read and approved the final manuscript.

## Funding

This work was supported by the Natural Science Foundation of China (grant no. 32060748), Fuxi Young Talents Fund of Gansu Agricultural University (GAUfx-04Y012), the Natural Science Foundation of China (grant no. 31960673), and Discipline Team Project of Gansu Agricultural University (No: GAU-XKTD-2022-20).

## Conflict of interest

The authors declare that the research was conducted in the absence of any commercial or financial relationships that could be construed as a potential conflict of interest.

## Publisher’s note

All claims expressed in this article are solely those of the authors and do not necessarily represent those of their affiliated organizations, or those of the publisher, the editors and the reviewers. Any product that may be evaluated in this article, or claim that may be made by its manufacturer, is not guaranteed or endorsed by the publisher.

## Footnotes

^1^ https://david.ncifcrf.gov/

## References

[1] Ermias E Yami A Rege J. Fat deposition in tropical sheep as adaptive attribute to periodic feed fluctuation. J Anim Breed Genet. (2002) 119:235–46. doi: 10.1046/j.1439-0388.2002.00344.x

[2] Kashan NEJ Azar GHM Afzalzadeh A Salehi A. Growth performance and carcass quality of fattening lambs from fat-tailed and tailed sheep breeds. Small Rumin Res. (2005) 60:267–71. doi: 10.1016/j.smallrumres.2005.01.001

[3] Lv FH Peng WF Ji Y Zhao YX Li WR Liu MJ. Mitogenomic meta-analysis identifies two phases of migration in the history of eastern Eurasian sheep. Molec Biol Evol. (2015) 32:2515–33. doi: 10.1093/molbev/msv139 26085518 PMC4576706

[4] Negussie E Rottmann OJ Pirchner F Rege J. Patterns of growth and partitioning of fat depots in tropical fat-tailed Menz and Horro sheep breeds. Meat Sci. (2003) 64:491–8. doi: 10.1016/S0309-1740(02)00227-9, PMID: 22063132

[5] Huang J Vieira A. DNA methylation, riboswitches, and transcription factor activity: fundamental mechanisms of gene–nutrient interactions involving vitamins. Mol Biol Rep. (2006) 33:253–6. doi: 10.1007/s11033-006-9005-y, PMID: 17077989

[6] Fraga MF Ballestar E Paz MF Ropero S Setien F Ballestar ML. Epigenetic differences arise during the lifetime of monozygotic twins. Proc Natl Acad Sci U S A. (2005) 102:10604–9. doi: 10.1073/pnas.0500398102, PMID: 16009939 PMC1174919

[7] Kaminsky ZA Tang T Wang SC Ptak C Oh GHT Wong AHC. DNA methylation profiles in monozygotic and dizygotic twins. Nat Genet. (2009) 41:240–5. doi: 10.1038/ng.286, PMID: 19151718

[8] Rideout III Eggan K Jaenisch R. Nuclear cloning and epigenetic reprogramming of the genome. Science. (2001) 293:1093–8. doi: 10.1126/science.1063206 11498580

[9] Huang X Chen YQ Xu GL Peng SH. DNA methylation in adipose tissue and the development of diabetes and obesity. Yi Chuan. (2019, 2019) 41:98–110. doi: 10.16288/j.yczz.18-206 30803941

[10] Zhang S Shen L Xia Y Yang Q Li X Tang G. DNA methylation landscape of fat deposits and fatty acid composition in obese and lean pigs. Sci Rep. (2016) 6:35063. doi: 10.1038/srep35063, PMID: 27721392 PMC5056348

[11] Hall IM. BEDTools: a flexible suite of utilities for comparing genomic features. Bioinformatics. (2010) 26:841–2. doi: 10.1093/bioinformatics/btq033 20110278 PMC2832824

[12] Lister R Pelizzola M Dowen R Hawkins D Hon G Tonti-Filippini J. Victor: human DNA methylomes at base resolution show widespread epigenomic differences. Nature. (2009) 462:315–22. doi: 10.1038/nature08514, PMID: 19829295 PMC2857523

[13] Straussman R Nejman D Roberts D Steinfeld I Blum B Benvenisty N. Developmental programming of CpG island methylation profiles in the human genome. Nat Struct Mol Biol. (2009) 16:564–71. doi: 10.1038/nsmb.1594, PMID: 19377480

[14] Kim HB Kumar A Wang L Liu GH Harris TE. Lipin 1 represses NFATc4 transcriptional activity in adipocytes to inhibit secretion of inflammatory factors. Mol Cell Biol. (2010) 30:3126–39. doi: 10.1128/MCB.01671-09, PMID: 20385772 PMC2876672

[15] Jiao X-L Jing J-J Qiao L-Y Liu J-H Li L-A Zhang J. Ontogenetic expression of Lpin2 and Lpin3 genes and their associations with traits in two breeds of Chinese fat-tailed sheep. Asian Austr J Anim Sci. (2016) 29:333–42. doi: 10.5713/ajas.15.0467 PMC4811783 26950863

[16] Mochida T Take K Maki T Nakakariya M Adachi R Sato K. Inhibition of MGAT2 modulates fat-induced gut peptide release and fat intake in normal mice and ameliorates obesity and diabetes in Ob/Ob mice fed on a high-fat diet. FEBS Open Bio. (2020) 10:316–26. doi: 10.1002/2211-5463.12778, PMID: 31837122 PMC7050258

[17] Zhao C Chen X Wu W Wang W Pang W Yang G. MAT2B promotes adipogenesis by modulating SAMe levels and activating AKT/ERK pathway during porcine intramuscular preadipocyte differentiation. Exp Cell Res. (2016) 344:11–21. doi: 10.1016/j.yexcr.2016.02.019, PMID: 26940012

[18] Ge CYJ Weber C Sun W Zhang H Zhou Y Cai C. The histone demethylase KDM6B regulates temperature-dependent sex determination in a turtle species. Science. (2018) 360:645–8. doi: 10.1126/science.aap8328 29748283

[19] Zhu C Li N Cheng H Ma Y. Genome wide association study for the identification of genes associated with tail fat deposition in Chinese sheep breeds. Biol Open. (2021) 10:bio054932. doi: 10.1242/bio.054932, PMID: 33942864 PMC8186729

[20] Bird A. DNA methylation patterns and epigenetic memory. Genes Dev. (2002) 16:6–21. doi: 10.1101/gad.947102 11782440

[21] Jaenisch R Bird A. Epigenetic regulation of gene expression: how the genome integrates intrinsic and environmental signals. Nat Genet. (2003) 33:245–54. doi: 10.1038/ng1089, PMID: 12610534

[22] Wang M Bissonnette N Dudemaine PL Zhao X Ibeagha-Awemu EM. Whole genome DNA methylation variations in mammary gland tissues from Holstein Cattle producing milk with various fat and protein contents. Genes. (2021) 12:1727. doi: 10.3390/genes12111727, PMID: 34828333 PMC8618717

[23] Jones PA. Functions of DNA methylation: islands, start sites, gene bodies and beyond. Nat Rev Genet. (2012) 13:484–92. doi: 10.1038/nrg3230, PMID: 22641018

[24] Horvath S. DNA methylation age of human tissues and cell types. Genome Biol. (2013) 16:1–5. doi: 10.1186/s13059-015-0649-6 PMC4015143 24138928

[25] Tian-fu G Zhi-yan Z Dong C Tian-Xiong Y Shi-Jun X Lu-sheng H. High resolution and single base genome-wide methylation variance analysis of muscle of large white pigs with different sexes. Acta Vet Zootech Sin. (2018) 49:2326–39. doi: 10.11843/j.issn.0366-6964.2018.11.004

[26] Wang K Ping-Xian WU Wang SJ Xiang JI Chen D Jiang AA. Epigenome-wide DNA methylation analysis reveals differentially methylation patterns in skeletal muscle between Chinese Chenghua and Qingyu pigs. J Integr Agric. (2022) 21:1731–9. doi: 10.1016/S2095-3119(21)63814-5

[27] Wang X Kadarmideen HN. An epigenome-wide DNA methylation map of testis in pigs for study of complex traits. Front Genet. (2013) 10. doi: 10.3389/fgene.2019.00405 PMC6502962 31114612

[28] Shao X Zhang C Sun MA Lu X Xie H. Deciphering the heterogeneity in DNA methylation patterns during stem cell differentiation and reprogramming. BMC Genomics. (2014) 15:978. doi: 10.1186/1471-2164-15-978, PMID: 25404570 PMC4242552

[29] Luo R Bai C Yang L Zheng Z Su G Gao G. DNA methylation subpatterns at distinct regulatory regions in human early embryos. Open Biol. (2018) 8:180131. doi: 10.1098/rsob.180131 30381360 PMC6223221

[30] Zhao CZ Wu HG Qimuge P WJ L. MAT2A promotes porcine adipogenesis by mediating H3K27me3 at Wnt10b locus and repressing Wnt/beta-catenin signaling. Biochim Biophys Acta Mol Cell Biol Lipids. (2018) 1863:132–42. doi: 10.1016/j.bbalip.2017.11.001, PMID: 29133280

